# Debunking the Myth of *Fusarium poae* T-2/HT-2 Toxin Production

**DOI:** 10.1021/acs.jafc.3c08437

**Published:** 2024-02-20

**Authors:** Thomas E. Witte, Carmen Hicks, Anne Hermans, Sam Shields, David P. Overy

**Affiliations:** † Agriculture and Agri-Food Canada, Ottawa Research and Development Centre, Ottawa, Ontario K1A 0C6, Canada; ‡ Department of Chemistry and Biomolecular Sciences, University of Ottawa, Ottawa, Ontario K1N 6N5, Canada

**Keywords:** *Fusarium poae*, T-2 toxin, trichothecenes, mycotoxins, Fusarium head blight, metabolomics

## Abstract

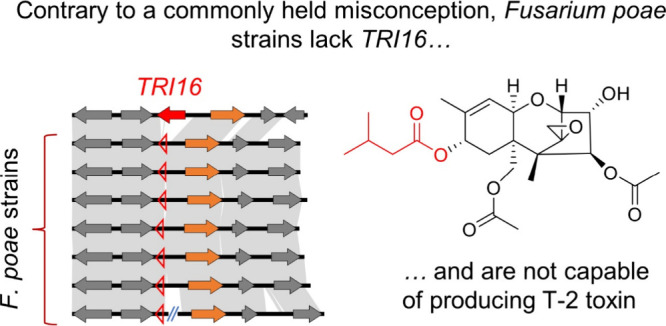

*Fusarium poae* is commonly detected in field surveys of *Fusarium* head blight (FHB) of cereal crops and can produce a range of trichothecene mycotoxins. Although experimentally validated reports of *F. poae* strains producing T-2/HT-2 trichothecenes are rare, *F. poae* is frequently generalized in the literature as a producer of T-2/HT-2 toxins due to a single study from 2004 in which T-2/HT-2 toxins were detected at low levels from six out of forty-nine *F. poae* strains examined. To validate/substantiate the observations reported from the 2004 study, the producing strains were acquired and phylogenetically confirmed to be correctly assigned as *F. poae*; however, no evidence of T-2/HT-2 toxin production was observed from axenic cultures. Moreover, no evidence for a *TRI16* ortholog, encoding a key acyltransferase shown to be necessary for T-2 toxin production in other *Fusarium* species, was observed in any of the *de novo* assembled genomes of the *F. poae* strains. Our findings corroborate multiple field-based and *in vitro* studies on FHB-associated *Fusarium* populations which also do not support the production of T-2/HT-2 toxins with *F. poae* and therefore conclude that *F. poae* should not be generalized as a T-2/HT-2 toxin producing species of *Fusarium*.

## Introduction

*Fusarium poae* is frequently isolated from *Fusarium* head blight (FHB)-damaged wheat, barley, and oat samples and is considered to be globally distributed.^1,2^ Often described as a “weak pathogen” in comparison to *F. graminearum*, *F. poae* infection during plant challenge experiments typically exhibits low levels of disease symptoms.^3−8^ Nevertheless, *F. poae* is a species of concern to cereal growers primarily because of its frequent detection in cereal samples and its ability to produce both type A and B trichothecene mycotoxins. *F. poae* trichothecene profiles from *in vitro* experiments are typically characterized by the presence of the type A trichothecenes diacetoxyscirpenol, monoacetoxyscirpenol, neosolaniol, and scirpentriol and the type B trichothecenes fusarenon-X and nivalenol (Table 1). Interestingly, in rare instances, subsets of *F. poae* isolates have been reported to produce the highly toxic type A trichothecenes T-2 and HT-2 toxins,^9,10^ which are of utmost concern to cereal producers because T-2 and HT-2 toxins are acutely toxic to consumers^11,12^ and are strictly regulated in feed.^13,14^ Given the gravity of the association of *F. poae* and T-2/HT-2 toxin production, and the rarity of observed production, it is imperative to critically evaluate previous claims of T-2/HT-2 production by *F. poae* strains.

**Table 1:** Summary of *F. poae* Trichothecene Profiling Results^a^

		no. of strains producing detected trichothecenes						
research study	*F. poae* strains	T-2/HT-2	DAS	MAS	FX	NIV	SCR	NEO
Thrane et al., 2004	49	6	46	45	38	41	33	7
Vogelgsang et al., 2008a	3	0	3	3	<3	3	–	<3
Kokkonen et al., 2010	3	0	0	–	–	–	–	0
Somma et al., 2010	81	0	59	–	59	69	–	62
Stenglein et al., 2014	5	0	–	–	–	5	–	–
Vanheule et al., 2017	61	0	59	–	16	12	–	54
O’Donnell et al., 2018	3	0	3	–	–	–	–	–
Witte et al., 2021	38	0	36	36	36	33	35	36
Kahla et al., 2023	2	0	1	–	–	–	–	0
***Totals***	**245**	**6**	**207**	**84**	**149**	**163**	**68**	**159**

a Abbreviations: DAS, 3,15-diacetoxyscirpenol; MAS, 15-monoacetoxyscirpenol; FX, fusarenon-X; NIV, nivalenol; SCR, scirpentriol; NEO, neosolaniol; −, not tested.

Trichothecenes are a diverse group of nonvolatile sesquiterpenoid natural products that cause deleterious effects on plant and animal cells through impairment of ribosomal peptide synthesis and mitochondrial function. Composed of highly functionalized 12,13-epoxytrichothecene scaffolds, trichothecenes are broadly classified as types A and B based on scaffold substitution profiles (detected in *Fusarium* spp.), or as types C and D by the presence of longer side chains which may be macrocyclized (not associated with *Fusarium* spp.). Trichothecene biosynthesis follows a multistep pathway involving enzymes encoded by *TRI* genes that are mostly clustered at a single genomic locus; however, three enzymes involved in tailoring trichothecene scaffolds are encoded by genes residing on two additional loci in many *Fusarium* species,^15^ including the *TRI101* locus and a two-gene *TRI1-TRI16* cluster which plays an essential role in the production of T-2/HT-2 toxins. Proposed biosynthetic routes for *Fusarium* trichothecenes have been extensively reviewed (for example, see ref (16)); with particular relevance to our current study, a critical biosynthetic step in the production of T-2/HT-2 toxins in *Fusarium* spp. involves the action of the TRI16 acyltransferase to acylate a hydroxyl at the C8 position of the trichothecene scaffold.

When comparing literature summarizing trichothecene production in *F. poae*, we noticed that the association of T-2/HT-2 toxin production with *F. poae* can be traced almost entirely to a single, influential study of secondary metabolites produced by *F. poae*, *F. sporotrichioides*, and *F. langsethiae* by Thrane et al.^9^ In that study, Thrane et al. focused on clarifying the mycotoxin profile of *F. langsethiae*, which at that time was only recently described, in contrast to *F. sporotrichioides* and *F. poae*, two other commonly detected species of head-blight associated Fusaria. Thrane et al.^9^ identified T-2 toxin associated trichothecenes (including T-2 toxin, HT-2 toxin and T-2 tetraol) in extracts of all *F. langsethiae* and *F. sporotrichioides* strains, as well as from a few of the *F. poae* strains examined (Table 1). The production of T-2/HT-2 toxins from *F. poae* was considered rare by Thrane et al.,^9^ detected in only 6/49 strains profiled, and was investigated by three independent laboratories, either via GC–MS/MS analysis of pentafluoropropionoinyl esterified derivatives (2 laboratories), or by GC electron capture detection of heptafluorbutyryl and trimethylsilyl derivatives (1 laboratory), in comparison to commercially available standards of T-2, HT-2, and T-2 tetraol toxins. Although the strains were grown on various media conditions by the different contributing laboratories, the published results neither specify which of the laboratories positively identified the presence of the toxins, on which media conditions the toxins were produced, nor is there reported biological replication employed in any experiments to confirm initial observations. Furthermore, beyond the labeling of “trace” amounts, no MS mass feature signal intensities were published, although the rarity of the *F. poae*-associated production of T-2 toxin is noted and described as “low amounts” by Thrane et al.^9^ Thrane et al.^9^ justifiably concluded that *F. langsethiae* and *F. sporotrichioides* are the primary culprits for T-2/HT-2 toxin production in the FHB disease complex.

What other historical evidence is there supporting the production of the T-2/HT-2 toxin by *F. poae*? In the time since the publication of Thrane et al.,^9^ subsequent mycotoxin profiling of reliably identified *F. poae* strains by multiple researchers has consistently reported the absence of T-2/HT-2 production from this species.^1,17−24^ To date, from literature accounts, 245 different strains of *F. poae* have been profiled for trichothecene content, with only the six strains identified by Thrane et al.^9^ being reported as T-2/HT-2 producers (Table 1). Although a few reports tentatively link *F. poae*-inoculated plants or *in vitro* media conditions with T-2 toxin detection, those reports describe unconvincingly “trace” T-2 or HT-2 signals observed at or below the limit of detection and were not reported on a per-strain basis,^25,26^ or the reports described field-sampling experiments seeking to correlate the quantitation of T-2/HT-2 in bulk homogenized field samples with that of the presence of *Fusarium* species’ DNA amplified from the bulk samples.^10,27^ Although this latter type of correlation analysis is a useful method for generating broad hypotheses linking toxins to groups of species within a complex such as FHB, the analysis of bulk homogenized field samples prohibits conclusive links between toxins and fungal species due to the frequent, in-field co-occurrence of *F. poae* with other known T-2 toxins producers, such as *F. langsethiae* or *F. sporotrichioides*. In contrast to these few studies, the majority of correlation analyses from cereal grain sampling surveys have consistently demonstrated that T-2/HT-2 detection is not positively correlated with the presence of *F. poae* DNA.^28−37^

The chemical profiling work published by Thrane et al.^9^ has been cited over 250 times in the past two decades, and of these, at least 43 citations attribute T-2 toxin production to the concept of *F. poae* as a species (based solely on observations from 6/49 strains reported in the Thrane et al. study). Additionally, many review articles and manuscript discussions simply refer to *F. poae* as a known T-2 toxin producer without providing any supporting evidence, even though in the study by Thrane et al.^9^ the reported occurrence of observed T-2/HT-2 toxin production in *F. poae* was limited to only 12% of the strains examined, or 2% of all *F. poae* strains profiled and reported in the literature to date (Table 1). The consistent absence of T-2/HT-2 production in strains of *F. poae* from numerous pathogenicity surveys and chemical screening has led multiple research groups to speculate that the reported T-2/HT-2-producing strains characterized by Thrane et al.^9^ were likely misidentified *F. langsethiae* isolates.^23,26,38−40^ This speculation is reasonable since *F. langsethiae*, previously referred to in the literature as “powdery poae” due to the macro- and microphenotypic similarity between the two species, is a known producer of T-2/HT-2 toxins. Indeed, any historical study of *F. poae* should be interpreted with caution, given that morphology alone cannot reliably distinguish this species from *F. langsethiae*, which was not formally described until 2004.^41,42^ Nevertheless, the *F. poae* strains from Thrane et al.^9^ were taxonomically supported by phylogenetics and by the simultaneous detection of other secondary metabolites such as beauvericin, a cyclic depsipeptide produced primarily by *F. poae* and not *F. langsethiae*, in reportedly T-2/HT-2 producing *F. poae* strain extracts. This level of support therefore weakens the “misidentification” speculation.

Given the importance of T-2 toxin contamination to agronomic trade and consumer health, we decided to further investigate the T-2 toxin-producing strains identified by Thrane et al.^9^ using a combination of targeted metabolomics and whole-genome sequencing. The two selected approaches were employed to confirm the chemical detection of T-2/HT-2 toxins (and relevant analogs) from strain extracts on a trichothecene-permissive medium, as well as the detection of *TRI16* orthologs within the genomes of the various strains that would support the possibility of T-2/HT-2 toxin production, even in the absence of detected chemical signals from the extracts.

## Materials and Methods

### Strain Selection

Five *F. poae* strains identified by Thrane et al.^9^ as T-2 and/or HT-2 producers (IBT 9924, IBT 9928, IBT 9976, IBT 9988, and IBT 400006) and a sixth strain reported to produce T-2 tetraol (IBT 9973) were requested from the IBT (Institut for Bioteknologi) strain collection at the Denmark Technical University Bioengineering department. All strains were successfully revived and maintained on Spezieller Nahrstoffarmer agar (SNA) plates, except for IBT 9976, which had lost viability in storage. The SNA medium formulation used consisted of 1 g of KH_2_PO_4_, 1 g of KNO_3_, 0.5 g of MgSO_4_·7H_2_O, 0.5 g of KCl, 0.2 g of glucose, 0.2 g of sucrose, and 20 g of agar per 1 L of distilled water.

Three additional strains were added to the analysis: *F. poae* strain *Fp*157 is a Canadian isolate whose genome and chemical phenotype was previously published,^23^*F. langsethiae* strain Fl201059 has been the subject of previous genetic and chemical profiling,^43^ and *F. sporotrichioides* strain *Fsp*184 is available at the Canadian Collection of Fungal Cultures (under accession DAOMC 238877).

### Metabolomics Protocol: Fermentation, Extraction, and UPLC–HRMS Analysis

The strain culturing, extract production, and data analysis protocols used in this study closely followed those published in Witte et al.,^22^ with the exception that only Yeast-Extract-Sucrose (YES) medium, a trichothecene-production eliciting medium, was used as a growth medium for the strains. The YES media formulation consists of 20 g of yeast extract, 150 g of sucrose, and 500 mg of MgSO_4_·7H_2_O per 1 L of distilled water. In brief, mycelium plugs were inoculated into slant tubes containing 15 mL of liquid YES medium in four replicates. After 14 days of growth at 25 °C in the dark, the mycelia were separated from the broth, and both broth and mycelium were frozen as separate samples. Samples were thawed, extracted in 15 mL of ethyl acetate, dried, and then resuspended in MS-grade methanol to a concentration of 500 μg/mL. The resuspended extracts were then analyzed on a Thermo Ultimate 3000 ultrahigh pressure liquid chromatograph coupled to a Thermo LTQ Orbitrap XL high resolution mass spectrometer (UPLC–HRMS). Reverse-phase chromatography was performed using a Phenomenex C18 Kinetex column (50 mm × 2.1 mm ID, 1.7 μm) running a gradient of water and acetonitrile exactly as published in Witte et al.,^22^ and mass spectrometry was performed in positive mode only.

### Metabolomics Data Analysis

The resulting high-resolution mass spectrometry RAW files were preprocessed using *MZmine* v2.59 or else directly examined in Thermo Qual Browser by plotting extracted ion chromatographs for each mass/charge ratio (*m*/*z*) and comparing them to standards processed under the same UPLC–HRMS methods. Within *MZmine2*, mass features were characterized by comparison to known retention times and exact *m*/*z* of mycotoxin standards, using the “Targeted feature detection” module with an intensity tolerance of 10%, a noise level of 3.0 E4, a *m*/*z* tolerance of 5.0 ppm, and a retention time tolerance of 0.08 min. Parameters were chosen based on careful examination of raw spectra including from methanol blanks processed every tenth sample, and uninoculated media extractions (YES media broth, extracted as described above) as controls. Mass feature annotation was enabled by comparison to commercial standards for T-2 toxin, HT-2 toxin, 3,15-diacetoxyscirpenol, neosolaniol, fusarenon-X, 15-monoacetoxyscirpenol, apicidin, aurofusarin, and beauvericin. If no standards were available, mass features were compared to features annotated from *F. poae* strain *Fp*157, including W-493 A/W-493 B, fusarin C, fusarin A, fusarin PM, and diacetoxynivalenol.^23^ Peak area values for the mass feature putatively annotated as fusarin C were selected to represent the “fusarin-associated” annotation. Other mass features matching the exact mass (<5 ppm) of T-2 toxin analogs, including 4-propanoyl T-2 toxin, 3-hydroxy T-2 toxin, and acetyl T-2 toxin, were tentatively annotated based on exact *m*/*z* matches, as no commercial standards were available for these features.

### Genomic DNA Purification and Sequencing

Genomic DNA (gDNA) for Illumina genome sequencing was generated by inoculating mycelium from *F. poae* isolates grown on SNA media into 250 mL Erlenmeyer flasks containing 50 mL first-stage media.^44^ The cultures were incubated at 25 °C and shaken at 180 rpm for 4 days. The mycelia were isolated by vacuum filtration, washed using sterile water, frozen in liquid nitrogen, and ground using a mortar and pestle until a fine powder was produced. Genomic DNA was extracted using a cetyltrimethylammonium bromide (CTAB) protocol with minor modifications including a scale up for a larger DNA yield, and a sodium acetate DNA precipitation in place of ammonium acetate.^45^ Isolated DNA was subjected to an RNase treatment post extraction and cleaned using the Genomic DNA Clean and Concentrator (Zymo Research, Irvine, CA).

The gDNA pellet was reconstituted in 200 μL of 10 mM Tris pH 8.0, and the gDNA concentration determined using a FLUOstar OPTIMA fluorometer (BMG LABTECH) and a PicoGreen dsDNA Quantitation Kit (Molecular Probes Inc.). The reconstituted gDNA was mechanically sheared to ∼300 bp fragments with a Covaris LE220 instrument and used as a template to construct PCR free Libraries with the NxSeq AmpFREE Low DNA Library kit (Lucigen) and TruSeq CD dual indices (Illumina) according to the Lucigen’s Library protocol. Indexed libraries were pooled, and sequencing was carried on a NextSeq500/550 (Illumina) using 2 × 150 bp NextSeq High Output Reagent Kit (Illumina) according to the manufacturer’s recommendations in order to obtain paired-end reads.

### Genome Assembly

Raw reads were trimmed of adapters, poor quality sites, and trailing *G*′s using fastp v0.23.2.^46^ Scaffold assembly was performed using SPAdes v3.10.1.^47^ The resulting assemblies were assessed for quality using Quast v5.0.2,^48^ and completeness was predicted via assessment of *Hypocreales*-associated benchmarked universal single copy ortholog (BUSCO) presence (4,494 orthologs from database *hypocreales_odb10*) using BUSCO v5.2.2,^49^ with gene models predicted using Augustus with *F. graminearum* preset training parameters.^50^

### Phylogenetic Analysis

All genomes not sequenced and assembled in this study were downloaded from the Genbank repository of nucleotide sequences. A total of 4,053 BUSCO genes (*Hypocreales_odb10* database) were detected as “single copy” and “complete” in all genomes using BUSCO v5.2.2,^49^ then aligned using MAFFT v7.470^51^ and trimmed with automated parameter detection using trimal v1.2.^52^ IQTREE v2.0 was used to infer phylogenetic relationships, using the Maximum Likelihood method with best model automatically determined per gene sequence using ModelFinder^53^ as part of the IQ-TREE v2.0.6 pipeline.^54^ Partition modeling was used, allowing genes to evolve under independent models.^55^ Bootstraps were calculated using both sh-LRT (*n* = 1000) and ultrafast values (*n* = 1000) and the tree was drawn to scale, with substitutions per site used to calculate branch lengths.^56^

### Gene Model Prediction and TRI16 Homologue Search

Gene models were predicted from repeat-masked, *de novo* assembled genomes using FUNANNOTATE v1.8.14.^57^ RepeatModeler v2.0.1^58^ was used to generate *de novo* libraries of repeated sequences for each genome, using the RepBase 2018 library of transposable elements (TE’s) to annotate TE’s, and RepeatMasker v4.2.1-p1 was used to softmask the assemblies in preparation for gene annotation. The FUNANNOTATE *predict* pipeline then used Evidence Modeler^59^ to weigh predicted models generated using BUSCO-supplied models to train Augustus v3.5.0 (with conserved gene models predicted from the sordariomycetes database),^49,50^ and GeneMark-ES.^60^*F. venenatum TRI16* (*FVRRES_00063*) nucleotide and amino acid sequences (XP_025587271.1) were used as queries to search for related genes in the *de novo* assemblies and predicted proteomes via BLASTn, tBLASTx, and BLASTp using default parameters within Geneious v 2022.2.2. Although *F. venenatum* has not yet been associated with T-2/HT-2 toxin production,^40^ it is more closely related to *F. poae* than other *TRI16*-containing species (queries using the *F. sporotrichioides TRI16* sequence gave similar results).

## Results and Discussion

### Taxonomic Confirmation of IBT Strains as *Fusarium poae*

**Figure 1:**
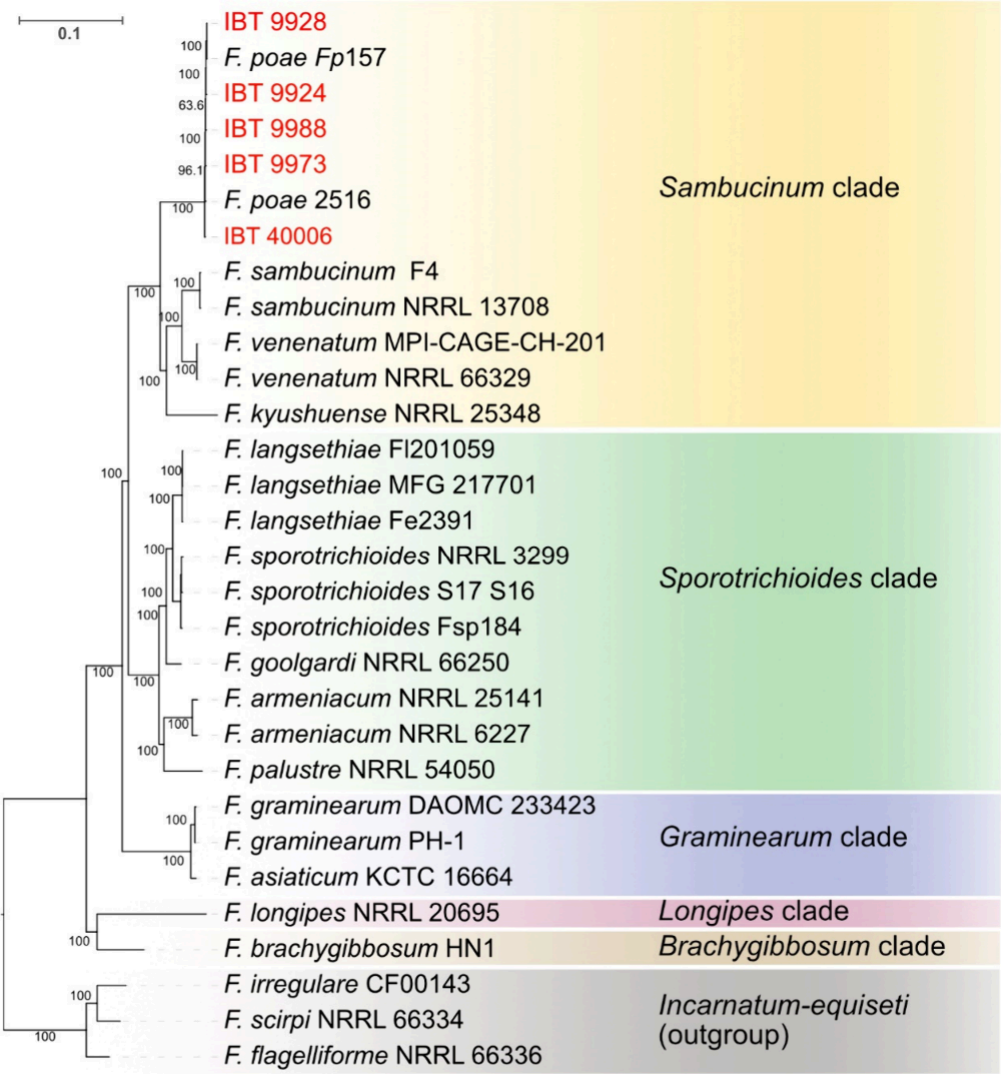
Maximum likelihood tree of representative isolates from clades within the *Fusarium sambucinum* species complex, built from nucleotide sequence alignments of 4,053 single copy orthologs (BUSCOs) from whole genome assemblies, including five strains sequenced in this study (red text). Representatives of the *F. incarnatum-equiseti* species complex were included as an outgroup. All IBT strains sequenced in this study cluster with *F. poae* strains, supporting their taxonomic assignment as *F. poae*. Numbers at nodes are bootstrap support values (SH-aLRT, *n* = 1000). Tree computed using IQTREE2.

Five strains of *F. poae* were reported to produce either T-2 toxin or HT-2 toxin, and a sixth strain was reported to produce T-2 tetraol.^9^ Of these strains, five were successfully revived (IBT 9924, IBT 9928, IBT 9973, IBT 9988, and IBT 40006), and a sixth (IBT 9976) was reported to have lost viability in storage. The five viable strains were whole-genome sequenced using Illumina Nextseq short-read sequencing to confirm their taxonomic relationship to previously genome sequenced *F. poae* isolates. All assembled genomes were assessed as high quality, at over 99.7% complete by analysis of *Hypocreales* benchmarked universal single-copy orthologs (BUSCOs), with an average length of approximately 38.6 Mb (Table 2). To infer evolutionary relationships, a phylogenetic cladogram was constructed using 4,053 BUSCOs modeled from the *F. poae* strains and a selection of relevant *Fusarium* species from the *Sambucinum* species complex, representing the *Sambucinum*, *Sporotrichioides*, *Graminearum*, *Longipes*, and *Brachygibbosum* clades, with three strains from the *F. incarnatum-equiseti* species complex included as an outgroup. The modeled BUSCOs incorporate 7,055,724 base pairs or approximately 20% of the total averaged *F. poae* assembly lengths and represent putative housekeeping or “core” gene coding sequences. Our analysis confirms all strains sequenced in this study were correctly assigned by Thrane et al.^9^ to a contemporary understanding of the *F. poae* taxon (Figure 1) and thereby refutes speculation that these were misidentified *F. langsethiae* (“powdery poae”) strains. Most are mating type 1–2, which is considered the rarer of the two mating types in profiled populations.^1,23^

**Table 2:** *Fusarium poae* Genome Statistics

Strain ID	IBT 9924	IBT 9928	IBT 9988	IBT 40006	IBT 9973
no. contigs (≥0 bp)	1729	1610	1701	1862	1725
no. contigs (≥1000 bp)	1180	1119	1206	1244	1179
Total length (≥0 bp)	38,587,486	38,642,078	38,591,118	38,557,541	38,447,436
GC (%)	47.33	47.28	47.43	47.58	47.14
N50	169,178	189,771	180,810	177,076	195,830
L50	68	57	63	66	60
no. N’s per 100 kbp	8.84	8.36	6.27	11.31	7.18
Estimated sequencing coverage (X)	133.6	169.6	132.9	121.9	131.5
BUSCO completeness (%)	99.8	99.7	99.7	99.7	99.7
Mating type	1–2	1–2	1–1	1–2	1–2
Genome accession	JASDAK010000000	JASDAL010000000	JASDAM010000000	JASDAN0100000000	JASDAO0100000000
SRA accession	SAMN34361090	SAMN34361091	SAMN34361092	SAMN34361093	SAMN34361094

### Targeted Metabolomics Analysis Does Not Support T-2 Toxin Production

**Figure 2:**
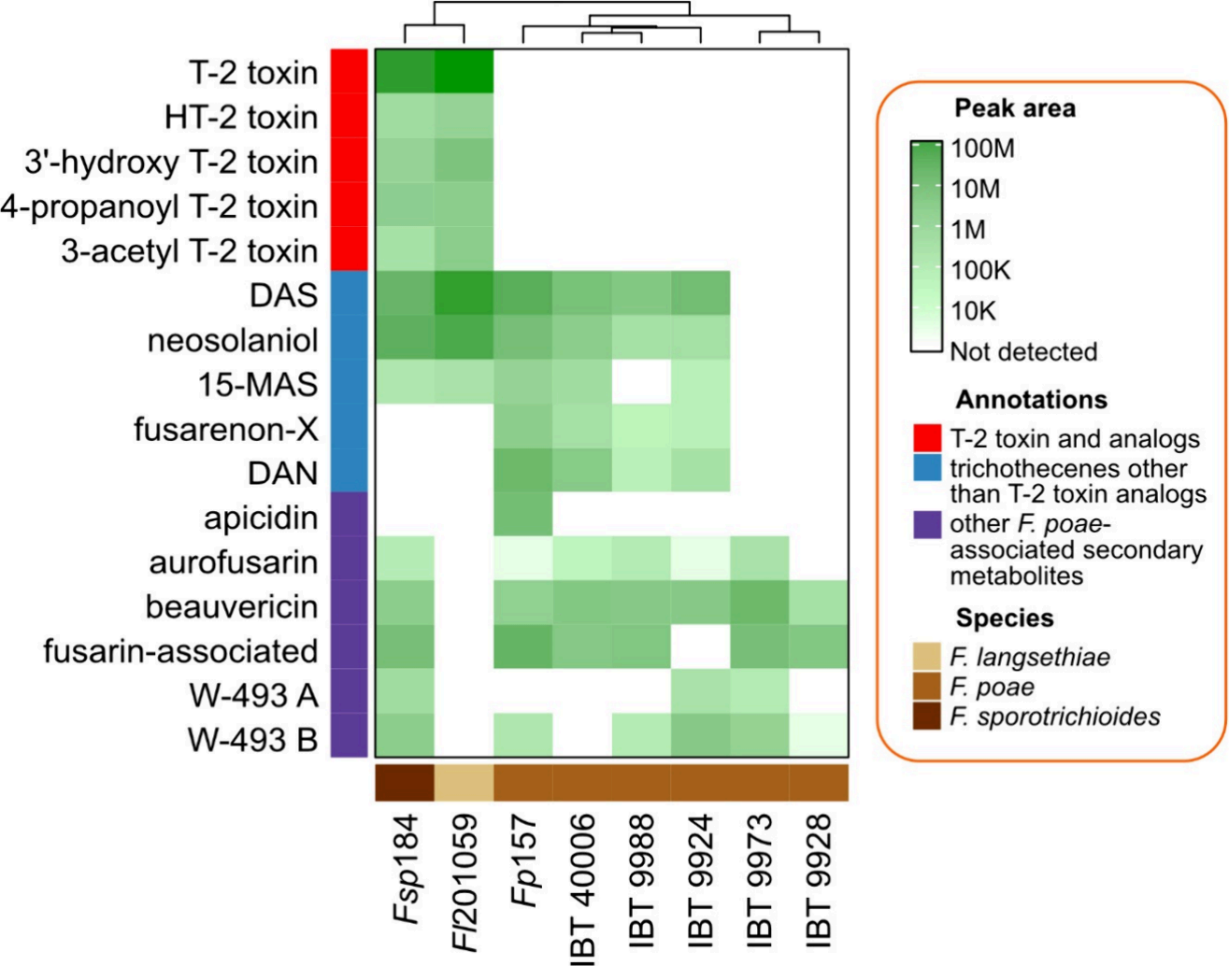
Targeted secondary metabolite profiles from UPLC-HRMS analysis of extracts from six *F. poae* strains, one *F. langsethiae* strain (Fl201059), and one *F. sporotrichioides* strain (*Fsp*184), cultured axenically on YES medium. Abbreviations: DAS, 3,15-diacetoxyscirpenol; 15-MAS, 15-monoacetoxyscirpenol; and DAN, diacetylnivalenol.

Organic extracts from axenically cultured strains were profiled for trichothecene content as well as all other secondary metabolites associated with *F. poae*, namely beauvericin, aurofusarin, fusarins, and W-493 A/B,^1,9,23^ using UPLC–HRMS. In addition to the five strains profiled from Thrane et al.,^9^ YES culture extracts from *F. poae* strain *Fp*157, *F. langsethiae* strain Fl201059, and *F. sporotrichioides* strain *Fsp*184 were also included in the targeted metabolomics analysis, allowing for confirmation that the culturing methods used were suitable for elicitation of T-2/HT-2 toxins from known producers (*F. langsethiae* and *F. sporotrichioides*). Our analysis indicated mass features associated with T-2/HT-2 toxins (including T-2 toxin and analogs HT-2 toxin, 4-propanoyl-T-2 toxin, 3′-hydroxy-T-2 toxin, and acetyl-T-2 toxin) were present in the *F. sporotrichioides* and *F. langsethiae* extracts but absent from all *F. poae* extracts (Figure 2). Moreover, the trichothecene profiles of most *F. poae* strains were consistent with known *F. poae* chemical phenotypes: specifically, diacetoxyscirpenol was detected as the primary trichothecene from all strains except IBT 9973 and IBT 9928, which had poor growth in YES media and did not produce any trichothecenes above the level of detection. Neosolaniol and fusarenon-X were detected from three strains, and 15-monoacetoxyscirpenol was detected from two strains. Neither nivalenol nor T-2 tetraol was detected in any of the strain extracts. This pattern is consistent with Vanheule et al.’s description of a “hierarchy” of *F. poae* trichothecene detection from *in vitro* culturing,^1^ wherein diacetoxyscirpenol is detected in greatest abundance from all TRI-producing strains, followed by increasingly infrequent detections of neosolaniol, fusarenon-X, and last nivalenol (if nivalenol is detected at all). The profiles are also consistent with Witte et al.’s profiling of Eastern Canadian *F. poae* strains.^23^ Other nontrichothecene *F. poae*-associated secondary metabolite mass features were also characterized, including beauvericin (5/5 strains), aurofusarin (4/5 strains), W-493B (4/5 strains), W-493 A (2/5 strains), and fusarin-associated metabolites (4/5 strains). Taken together, all chemical phenotypes of the strains profiled in Thrane et al.^9^ are consistent with known *F. poae* chemical phenotypes, even in the cases where trichothecenes were not detected, suggesting all five IBT strains are all nonexceptional *F. poae* strains and are not T-2 toxin producers under the conditions tested.

### An Intact TRI16 Gene Is Absent in All IBT *F. poae* Genomes

**Figure 3:**
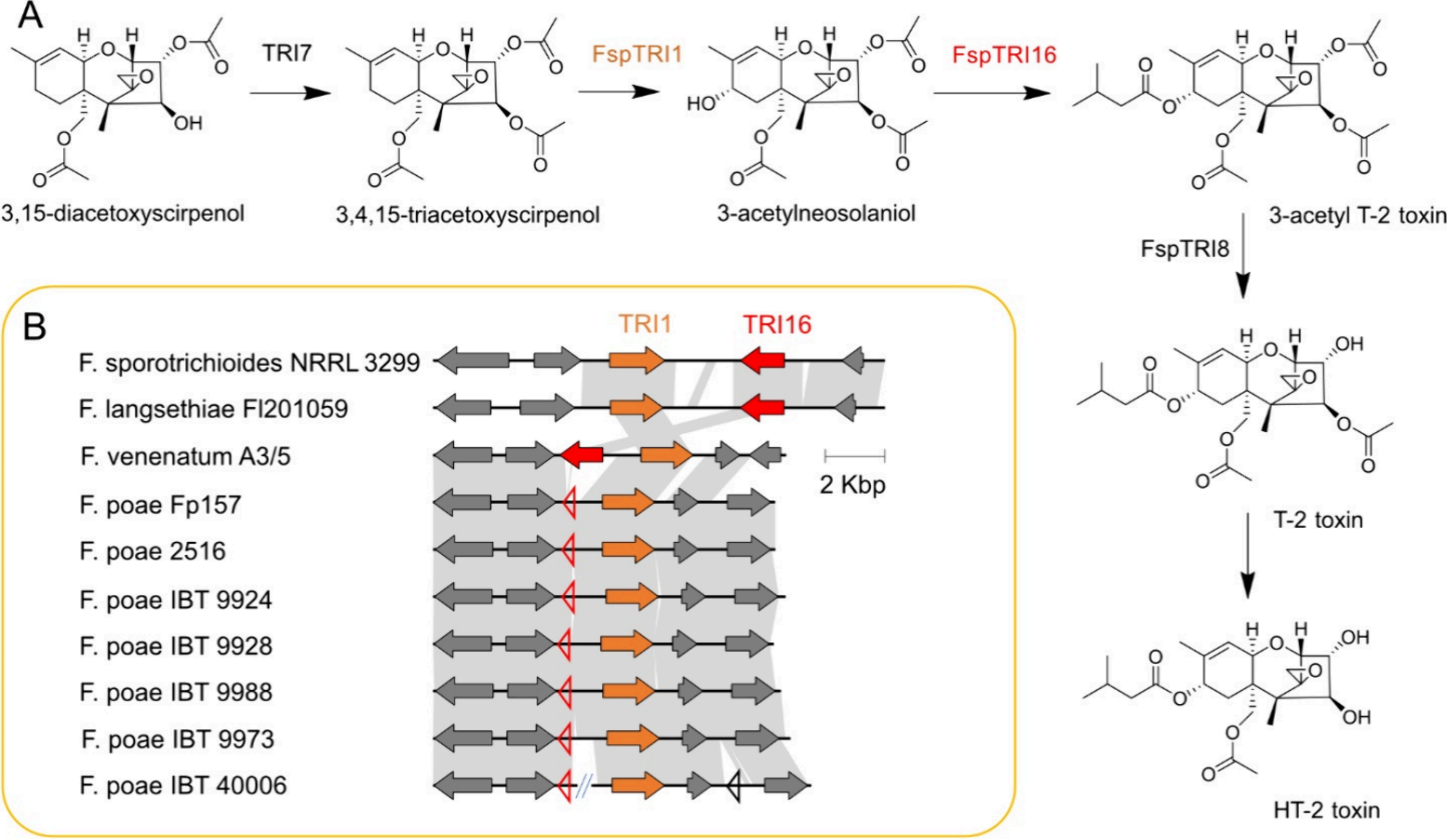
(A) Diagram of the final biosynthetic steps of the production of T-2 toxin by *F. sporotrichioides*, adapted from McCormick et al.^16^ Proposed biosynthetic steps involved in the production of neosolaniol and fusarenon-X, also detected from *F. poae* extracts, include alternative degrees of TRI1-mediated oxidation of carbons 7 and 8, and are not shown here. (B) Comparison of the two-gene *TRI1*–*TRI16* biosynthetic gene cluster neighborhood in relevant *Fusarium* species, including all *F. poae* strains profiled in this study, *F. poae* strains 2516 and *Fp*157, *F. venenatum* strain A3/5, and strains from T-2 toxin producing species *F. langsethiae* and *F. sporotrichioides*. Hollow triangles indicate pseudogenized remnants of *TRI16* in *F. poae* genomes and one other pseudogene in the neighborhood of *TRI1* in IBT 40006. The *F. poae* IBT 40006 genome was also fragmented at a region of low GC content immediately adjacent to the *TRI1* gene and *TRI16* remnant (relevant contigs were concatenated for the synteny analysis).

Recognizing that strain degeneration can occur due to prolonged periods of cryostorage (20+ years in this case) and might therefore lead to irreproducibility of the results observed by Thrane et al.,^9^ we searched the genomes of the IBT *F. poae* strains for the presence of homologues of the acyltransferase-encoding gene *TRI16*, expression of which is required for T-2/HT-2 metabolite production^61^ (Figure 3A). Whole-genome assemblies were generated for all of the IBT strains to account for the possibility of potential *TRI16* homologue sequence divergence (Table 2). This approach was particularly relevant for *F. poae* since recent genomic and metabolomic studies of *F. poae* isolates have identified small, strain-specific and transcriptionally active chromosomes associated with the evolution of novel chemical phenotypes, termed “supernumerary” or “accessory” chromosomes,^23,62,63^ on which a potential *TRI16* homologue could reside and would account for unique trichothecene production in a subpopulation of *F. poae*. Each of the genomes were therefore assembled *de novo* rather than aligning to a reference assembly, and our *TRI16*-homologue search was broadened to include divergent genes with predicted acyltransferase function.

The results of our genomic profiling were consistent with previous analyses exploring the evolution of trichothecene biosynthesis in *Fusarium*:^15^*F. poae* isolates likely all evolved from a common ancestral lineage in which the *TRI16* gene appears alongside *TRI1* in a two-gene cluster. However, in all *F. poae* strains sequenced so far, including the five published in this study, Belgian isolate 2516, and Canadian isolate *Fp*157, *TRI16* has been pseudogenized via truncation, while *TRI1* appears intact (Figure 3B). This pattern was also confirmed in 58 additional unpublished *F. poae* genomes from Canadian isolates (data not shown). The truncated *TRI16 pseudogene* sequence appears as an approximately 445 base-pair fragment of the 3′-terminus of the gene, whereas the functional *TRI16* gene is approximately 1,450 base pairs long in closely related species *F. sporotrichioides*, *F. langsethiae*, and *F. venenatum*. Apart from the truncated *TRI16* pseudogene, no other genes with notable homology (minimum 30% amino acid identity over 70% of the query sequence, see Supporting Information Tables S1 and S2) to *F. sporotrichioides* or *F. venenatum TRI16* was detected anywhere in the assembled *F. poae* genomes/proteomes. BLASTp searches querying the amino acid sequence of *F. venenatum TRI16* against the predicted proteomes of the *F. poae* strains matched only very distantly related orthologs which are present in all *F. poae* genomes sequenced to date and are not associated with trichothecene C8 acylation. The nearest detected relative of *F. venenatum TRI16* in *F. poae* is *TRI101* (20.4% nt identity over 98% of the query sequence), which is consistent with previously established *TRI16*/*TRI101* evolutionary relationships.^61^ Taken together, the results strongly indicate that accessory genome-associated *TRI16* orthologues are absent in this population. Other putative acyltransferases encoded for in genomes examined were both unrelated to *TRI16* and were present in all *F. poae* genomes published to date (as well as the 58 unpublished genomes we have generated thus far), suggesting that their presences are not specific to the reportedly T-2/HT-2 toxin producing strains from Thrane et al.^9^ Therefore, it is highly unlikely that the distantly related acyltransferase homologues convergently evolved to encode for enzymes that will acylate the 3-acetylneosolaniol C8 hydroxyl with isovalerate to produce T-2/HT-2 toxins in the strains profiled by Thrane et al.^9^

### Evidence Is Lacking for the Production of T-2 or HT-2 Toxins by *F. poae*

Precedence in the scientific literature for T-2/HT-2 toxin production as attributed to *F. poae* is associated with a select few *F. poae* strains, production of which was reported as rare and of low abundance by Thrane et al.^9^ Taken as a whole, the genetic and metabolomic evidence presented in our current study indicates that the *F. poae* strains profiled by Thrane et al.^9^ are not able to produce T-2 toxin or related analogs. We have confirmed the strains were not misidentified, as supported by whole genome-based phylogenetic analyses and chemical phenotypes, which were consistent with recent work profiling European and Canadian *F. poae* populations. It is therefore difficult to explain the initial reports of T-2/HT-2 production from said strains. The possibility that these strains once had the capacity to make T-2 associated toxins, due to the presence of a *TRI16* homologue (possibly in an accessory region or plasmid), is unlikely since all five strains would have had to have simultaneously lost this ability while in storage. The mycotoxin profiles detected in this study are consistent with the presence of a trichothecene biosynthetic pathway that progresses no further than neosolaniol production. Given that neosolaniol is the last biosynthetic precursor to T-2/HT-2 toxins and involves activation of *TRI1*, the genetic neighbor of *TRI16* needed for T-2/HT-2 toxin production, we can reasonably conclude that all homologous biosynthetic gene clusters associated with T-2/HT-2 toxin production in *F. sporotrichioides* are activated in the trichothecene-producing *F. poae* strains included in this study, and the resulting lack of T-2/HT-2 toxin production indicates the strains are in fact not capable of T-2/HT-2 production.

Without a more detailed accounting of the work of Thrane et al.,^9^ including the diagnostic mass feature signal intensities observed, experimental replication/validation, media conditions tested, and specificity of which laboratories reported positive detection of the T-2/HT-2 toxins for the *F. poae* strains, it remains difficult to speculate on why these strains were associated with low abundance T-2/HT-2 toxin production. Nevertheless, given the widespread occurrence of *F. poae* in FHB surveys, the extreme toxicity of T-2 associated mycotoxins, and the lack of metabolomic and genomic evidence for T-2/HT-2 toxin production from any *F. poae* strain profiled to date, we believe it is important at this time to decouple the association of the species *F. poae* with the production of the T-2/HT-2 toxin. We reiterate that there has been no convincing proof that any *F. poae* strain produces T-2/HT-2 toxins, and moving forward, we consider it incumbent on future *F. poae* researchers to conclusively prove a strains’ capacity to produce T-2/HT-2 toxin if they intend to generalize the *F. poae* species as such a producer. Additionally, we note that our assertions do not diminish the need to further study *F. poae*, as it is a proven producer of trichothecene mycotoxins, which include diacetoxyscirpenol, nivalenol, and fusarenon-X, each of which are demonstrably toxic to consumers and should be of concern to cereal producers, particularly of oats.

## Data Availability

Assembled genomes and raw reads were published at the NCBI Genbank/SRA under bioproject ID PRJNA961673. All assembly versions included in this study are the first versions.

## Supporting Information Available

tblastx results of TRI16 search; blastp results of TRI16 search (PDF)
