# Supplementary material for: Debunking the Myth of Fusarium poae T-2/HT-2 Toxin Production
Source: J Agric Food Chem. 2024 Feb 20;72(8):3949–57. doi: 10.1021/acs.jafc.3c08437 (PMC10905990; doi:10.1021/acs.jafc.3c08437)
Supplement: Supplementary file 1 — jf3c08437_si_001.pdf [file jf3c08437_si_001.pdf]

Supporting Information

Debunking the myth of *Fusarium poae* T-2/HT-2 toxin production.

Thomas E. Witte[1,2]; Carmen Hicks[1]; Anne Hermans[1]; Sam Shields[1]; David P. Overy[1,*]

[1]Agriculture and Agri-Food Canada, Ottawa Research and Development Centre, K1Y 4X2, Ottawa, Ontario, Canada.

[2]Department of Chemistry and Biomolecular Sciences, University of Ottawa, K1N 6N5 Ottawa, Ontario, Canada.

* Corresponding author, david.overy@agr.gc.ca

Table S1. tblastx results comparing *F. venenatum tri16* nt sequence to whole genome assemblies from IBT strains sequenced in this study (and Fp157, previously published). Values are % nt ID match / % query coverage

	IBT9924	IBT9928	IBT9973	IBT9988	IBT40006	Fp157	Gene ID from Fp157	# of *F. poae* genomes with matching hit (n=58)
hit1	20.4/96.9	20.4/96.9	20.4/96.9	20.4/96.9	20.4/96.9	20.4/96.9	*FPOAC1_011686* (*tri101*)	58
hit2	64.0/10.4	62.0/10.4	64.0/10.4	64.0/10.4	62.0/10.4	62.0/10.4	none (*tri16* pseudogene)	58
hit3	20.7/64.3	20.7/64.3	20.7/64.3	20.7/64.3	20.7/64.3	20.7/64.3	*FPOAC1_005352*	58
hit4	25.1/33.4	25.1/33.4	25.1/33.4	25.1/33.4	23.3/33.4	25.1/33.4	*FPOAC1_004207*	58
hit5	23.3/33.4	23.3/33.4	23.3/33.4	23.3/33.4	24.7/33.4	23.3/33.4	*FPOAC1_006138*	58

Table S2. blastp results comparing *F. venenatum tri16* aa sequence to predicted proteomes from IBT strains assembled in this study (and Fp157, previously published) Values are % aa similarity match / % query coverage

	IBT9924	IBT9928	IBT9973	IBT9988	IBT40006	Fp157	Gene ID from Fp157
hit1	53.2/96.9	53.2/96.9	53.2/96.9	53.4/96.9	53.2/96.9	53.2/96.9	*FPOAC1_011686* (*tri101*)
hit2	54.5/64.1	54.5/64.1	54.5/64.1	54.5/64.1	54.6/64.3	54.5/64.1	*FPOAC1_005352*
hit3	56.5/33.4	56.5/33.4	56.5/33.4	56.5/33.4	56.5/33.4	56.5/33.4	*FPOAC1_004207*
hit4	53.5/33.4	53.5/33.4	53.5/33.4	53.5/33.4	49.2/98.7	53.5/33.4	*FPOAC1_006138*
hit5	50.4/66.6	50.1/66.6	50.7/66.6	50.7/66.6	50.7/66.6	50.1/66.6	*FPOAC1_012818*
